# A Model-Assisted Probability of Detection Framework for Optical Fiber Sensors

**DOI:** 10.3390/s23104813

**Published:** 2023-05-16

**Authors:** Francesco Falcetelli, Nan Yue, Leonardo Rossi, Gabriele Bolognini, Filippo Bastianini, Dimitrios Zarouchas, Raffaella Di Sante

**Affiliations:** 1 Department of Industrial Engineering—DIN, University of Bologna, 47121 Forlì, Italy; raffaella.disante@unibo.it; 2 Department of Aerospace Structures and Materials, Faculty of Aerospace Engineering, Delft University of Technology, 2629 HS Delft, The Netherlands; n.yue@tudelft.nl; 3 IMM Institute, Consiglio Nazionale delle Ricerche, 40129 Bologna, Italy; rossi@bo.imm.cnr.it (L.R.); bolognini@bo.imm.cnr.it (G.B.); 4 SOCOTEC Photonics, 40069 Zola Predosa, Italy; filippo.bastianini@socotec.com; 5 Center of Excellence in Artificial Intelligence for Structures, Prognostics & Health Management, Aerospace Engineering Faculty, Delft University of Technology, Kluyverweg 1, 2629 HS Delft, The Netherlands; d.zarouchas@tudelft.nl

**Keywords:** MAPOD, optical fiber sensors, probability of detection, distributed sensing, structural health monitoring

## Abstract

Optical fiber sensors (OFSs) represent an efficient sensing solution in various structural health monitoring (SHM) applications. However, a well-defined methodology is still missing to quantify their damage detection performance, preventing their certification and full deployment in SHM. In a recent study, the authors proposed an experimental methodology to qualify distributed OFSs using the concept of probability of detection (POD). Nevertheless, POD curves require considerable testing, which is often not feasible. This study takes a step forward, presenting a model-assisted POD (MAPOD) approach for the first time applied to distributed OFSs (DOFSs). The new MAPOD framework applied to DOFSs is validated through previous experimental results, considering the mode I delamination monitoring of a double-cantilever beam (DCB) specimen under quasi-static loading conditions. The results show how strain transfer, loading conditions, human factors, interrogator resolution, and noise can alter the damage detection capabilities of DOFSs. This MAPOD approach represents a tool to study the effects of varying environmental and operational conditions on SHM systems based on DOFSs and for the design optimization of the monitoring system.

## 1. Introduction

Optical fiber sensors (OFSs) have long proven to outperform conventional strain gauges and, more in general, equivalent electrical strain sensors. Their advantages comprehend long durability, high accuracy, immunity to electromagnetic fields, and multiplexing capabilities [1]. Moreover, their light weight and small size make OFSs ideal candidates to be embedded into composite laminates [2,3,4] and additive manufacturing structures [5].

Distributed OFSs (DOFSs) inherit the advantages of traditional OFSs, such as fiber Bragg gratings (FBGs) [6,7], but offer the advantage of the increasing number of available sensing elements.

DOFSs typically rely on three scattering phenomena, namely Raman, Brillouin, and Rayleigh scattering [8]. Raman scattering is mainly used for temperature measurements, whereas Brillouin and Rayleigh scattering is mainly employed to measure both strain and temperature [9]. Brillouin scattering traditionally offers higher sensing ranges and lower spatial resolutions compared to Rayleigh scattering [8]. For this reason, the former is the preferred solution for most civil engineering applications, whereas the latter is the most suited for monitoring smaller structures typical of aerospace and automotive engineering.

Despite the high potential of this technology, the technology readiness level (TRL) of DOFSs in SHM is still surprisingly low. From a structural health monitoring (SHM) perspective, DOFSs cannot be used without a rigorous methodology that certifies their damage detection performance. Indeed, the lack of such a certification protocol prevents the successful widespread of DOFSs. DOFSs share this problem with other SHM technologies [10], such as guided Lamb waves (GLW) and acoustic emissions (AEs) [11].

On the other hand, the successful implementations of some mature non-destructive evaluation (NDE) methods are supported by a rigorous performance assessment framework based on probability of detection (POD) curves, as described in the MIL-HKBK-1823A [12]. SHM methods must demonstrate an equivalent or superior performance compared to NDE methods in order to motivate industrial adoption.

The SHM community tried to apply the same approach to SHM, but there are intrinsic differences between NDE and SHM. First, SHM systems are more difficult and expensive to manufacture, making reaching a statistically significant number of tests unfeasible. Second, the permanently installed sensing system in the structure makes the SHM system susceptible to varying environmental and operational conditions (EOCs). Third, SHM data suffer from spatial and temporal correlation, which infringes the independence of the observation hypothesis, a cornerstone for the linear regression models used for building POD curves [13,14].

Meeker et al. were among the first to present this problem to the SHM community [15]. They proposed handling SHM data with alternative statistical models, such as the length at detection (LaD) method and the random effect model (REM) [16]. These techniques have been implemented in a few SHM applications but never for DOFSs.

Falcetelli et al. proposed an experimental methodology based on the LaD method to qualify the detection performance of DOFSs for delamination detection into double-cantilever beam (DCB) specimens under quasi-static loading conditions [17]. The results were promising but simultaneously raised new questions and challenges. For example, is it possible to upscale the results obtained at a coupon level to a higher-level component using a building block approach? Is it possible to estimate the effect of varying EOCs on POD curves for DOFSs? How can we coexist with the scarcity of data typical of SHM systems?

These scientific questions epitomize the motivation behind this paper, the need for a model-assisted POD (MAPOD) approach applied to DOFSs. In other words, a MAPOD framework consists of a methodology to construct POD curves with the aid of a numerical model. In the NDE community, MAPOD approaches have already been developed [18,19,20], and there are already software programs [21,22] capable of producing POD curves for different NDE techniques. For the SHM community, however, this topic is relatively new and still in its infancy [23]. There are only a few studies that tried to develop such a MAPOD framework and only for specific SHM techniques mainly based on guided Lamb waves (GLW) [24,25] and bulk-wave ultrasonic sensors [26]. For acoustic emission (AE) analyses, modeling a real AE event is difficult due to its broadband nature [27]; therefore, only experimental approaches based on the Hsu–Nielsen source are used [28,29].

Sbarufatti et al. proposed a MAPOD framework for the performance qualification of an SHM system based on FBGs for the fatigue crack monitoring of helicopter fuselage panels and a helicopter tail boom [30,31].

The authors would like to stress that a MAPOD approach has never been applied to DOFSs and represents the most remarkable element of novelty of this research.

A MAPOD approach for DOFSs needs to account for all factors that influence the performance of DOFSs. The structure geometry and type of loading are determinant factors on POD due to the nature of strain sensing. In a recent article [17], the authors demonstrated that the DOFSs perform better for mode I delamination detection when the structure is loaded in quasi-static conditions compared to fatigue loading. Indeed, fatigue loading conditions are usually associated with high noise values. Moreover, delamination growth occurs at lower loads than the static case, resulting in a lower signal-to-noise ratio (SNR) from a DOFS perspective.

Moreover, depending on the laminate characteristics, the strain field in the process zone can be different [32]. Stutz et al. show that fiber bridging modifies the expected strain value in the process zone, which affects the damage index used to build POD curves [33]. Therefore, from a MAPOD point of view, having a high-fidelity model of the structure, loading conditions, and damage is imperative.

Another important yet often overlooked factor of optical strain sensing is the strain transfer effect from the structure to the fiber core [34,35,36]. Indeed, the strain in the structure under investigation requires a certain fiber length to be transferred entirely to the sensing element of the optical fiber, the core. This delay results from the shear lag theory and depends on the mechanical and geometrical properties of the DOFS and the adhesive. Therefore, the DOFS must be modeled to incorporate the strain transfer effect into the model [34]. The proposed MAPOD approach links the strain transfer problem to the damage detection problem, another significant element of novelty in this research. Indeed, both the strain transfer and damage detection with DOFSs were intensely studied in the literature, but their reciprocal implications, namely, the effects of the strain transfer in POD curves, have never been investigated.

Measuring itself brings extra uncertainty in the system depending on the interrogator unit and the operator experience [37]. This research article analyzes and discusses all these aspects of the measurement chain.

The article is structured as follows: the section “Material and Methods” displays the model architecture; the section “The DCB case study” considers the DCB case study to validate the model; the section “Results” shows how POD curves depend on the model parameters; the section “Discussion” reflects on the possibility to upscale the MAPOD methodology to a real aerospace structure; and the section “Conclusions” summarizes the central aspect of the study and provides suggestions for future research.

## 2. Materials and Methods

### 2.1. MAPOD Concept for DOFSs

**Figure 1:**
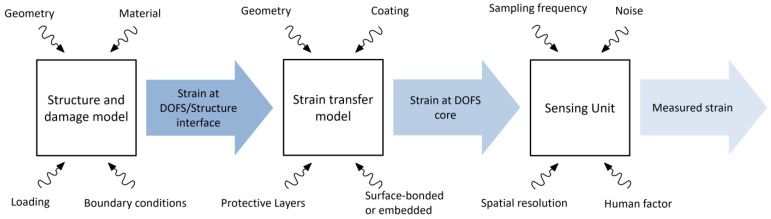
Flowchart of the proposed MAPOD approach.

Several factors affect the performance of DOFSs for damage detection: the structure geometry and type of loading, the strain transfer from the structure to the fiber core, and the measurement process [17]. Therefore, each one of these variables should be adequately modeled in a MAPOD framework. Figure 1 summarizes these steps in a flowchart, highlighting the main variables affecting the final measured strain.

First, the model of the structure and damage propagation is required. This model aims to reconstruct the strain at the DOFS/structure interface. One can leverage an existing analytical model based on physics; however, finite element analysis (FEA) might be required for complex geometries. Finally, if the structure or damage propagation is too complex for modeling, one can rely on data-driven models based on experimental data. Regardless of the approach used for modeling the structure, the MAPOD framework requires the user to define a spatial domain vector x, representing the extent of the segment of DOFS used for monitoring (see Equation (1)).

(1) $$\;x\;=\left[x_{0},\cdots ,x_{N}\right]\;$$

The spatial domain is discretized with a user-defined spatial resolution Δx, which must be sufficient to resolve the smallest geometrical feature.

Second, the discrepancy between the strain at the DOFS/structure interface and the DOFS core should not be neglected. This discrepancy can be considered by introducing strain transfer models [34].

Third, the type of interrogation unit affects the final measured strain and must be considered. Within the key variables, particular attention should be given to the human factor, which is often neglected but is of paramount importance. The interrogator resolution can be determinant in certain conditions as well as the signal-to-noise ratio (SNR).

### 2.2. Strain Transfer

The strain transfer mechanism plays a crucial role in measurement accuracy. Regardless of the working principle, optical fiber sensors measure the deformation of their core, not the deformation of the structure underneath or around it. Therefore, the strain profile at the optical fiber core is a distorted version of the original strain profile present in the structure to be monitored.

The general solution to the strain transfer problem considering a uniform strain field is given by Equation (2) [34,38,39,40,41]:

(2) $$\frac{d^{2}\varepsilon_{f}}{dx^{2}}-k^{2}\varepsilon_{f}=-k^{2}\varepsilon_{s}$$

where $\varepsilon_{f}$ is the strain vector of the optical fiber core, the symbol $\varepsilon_{s}$ is the input strain vector of the structure, and (k) is the shear lag parameter, condensing the mechanical and geometrical properties of the system.

Equation (2) is a second-order linear non-homogeneous differential equation with constant coefficients, whose solution is given by Equation (3):

(3) $$\varepsilon_{f}\left(x\right)=C_{1}e^{-kx}+C_{2}e^{kx}+\varepsilon_{s}$$

The integration constants $C_{1}$ and $C_{2}$ can be determined by applying the corresponding boundary conditions. It is possible to simulate the optical fiber response to a step input of intensity $\varepsilon_{s}$, by imposing the following boundary conditions:

(4) $$\left\{\begin{array}{l} \varepsilon_{f}\left(0\right)=0\;\\ \varepsilon_{f}\left(x\to +\infty \right)=\varepsilon_{s} \end{array}\right.$$

It follows that the integration constants are

(5) $$\left\{\begin{array}{l} C_{1}=0\;\\ C_{2}=-\varepsilon_{s} \end{array}\right.$$

Therefore, the step response of the system is given by Equation (6):

(6) $$\varepsilon_{f}\left(x\right)=\varepsilon_{s}\left(1-e^{-kx}\right)$$

It is convenient to define a characteristic space constant γ, defined as

(7) $$\gamma =\frac{1}{k}$$

In this way, Equation (6) can be rewritten as

(8) $$\varepsilon_{f}\left(x\right)=\varepsilon_{s}\left(1-e^{-\frac{x}{\lambda }}\right)$$

Equation (8) resembles the step response of a first-order system where the space variable *x* substitutes the time variable t, and the time constant, usually denoted with τ, is substituted with the characteristic space constant λ, representing the length required to reach approximately 63% of the strain present in the structure.

Therefore, it is possible to define the positive side (x>0) of the system transfer function as

(9) $$\Gamma^{+}\;\left(x\right)=\frac{1}{\lambda }e^{-\frac{x}{\lambda }}$$

Exploiting the symmetry that should have the transfer function (strain can also propagate backward), it is possible to extend the system transfer function to negative values, which, after normalization, leads to Equation (10):

(10) $$\Gamma \left(x\right)=\frac{1}{2\lambda }e^{-\frac{x}{\lambda }}$$

This result represents the system response to a unit impulse $\delta \left(x\right)$ and coincides with the mechanical transfer function proposed by Billon et al., who modeled the strain profile response induced by a surface crack [42]. At this point, it is possible to compute the strain field in the DOFS core, resulting from an arbitrary strain field in the structure $\varepsilon_{s}\left(x\right)$, using Equation (11):

(11) $$\varepsilon_{f}\left(x\right)=\left(\Gamma ⊗\varepsilon_{s}\right)\left(x\right)$$

Moreover, the characteristic space constant λ is assumed to vary across the different specimens to take into account the inherent variability associated with the limited repeatability of the bonding process and the possible non-homogeneity of the DOFS coating. Therefore, the characteristic space constant of the ith specimen is sampled from a normal distribution with a $\mu_{\lambda }$ mean and a $\sigma_{\lambda }$ standard deviation:

(12) $$\lambda_{i}\sim N\left(\mu_{\lambda },\sigma_{\lambda }\right)$$

In a previous study, Falcetelli et al. developed a methodology to compute $\mu_{\lambda }$ [34]. This strain transfer model, similar to analogous models available in the literature, requires accurate knowledge of the geometrical and mechanical properties of the optical fiber, the adhesive, and the host structure. However, in most cases, these properties are not available with the required degree of accuracy. Therefore, a more practical approach would be to assess the value of $\mu_{\lambda }$ by performing a tensile test with the DOFS bonded in the specimen surface and fitting the chosen analytical model.

Then, by repeating the procedure several times using different specimens, one can improve the accuracy of $\mu_{\lambda }$ and assess $\sigma_{\lambda }$, thus incorporating the variability associated with the bonding process in the model. It is also interesting to notice that in this MAPOD model, the shear lag constant is considered constant within the specimen, i.e., λ does not change along the fiber axis but only between different specimens. This assumption holds in cases where the bonding or the embedding is homogeneous without particular geometrical variations along the fiber path. If the fiber is bonded along a longer path, it is reasonable to assume that changes in λ can also occur within the same specimen. Nevertheless, in this MAPOD study, it is hypothesized that λ does not change along the fiber length for simplicity.

### 2.3. Interrogator Resolution

The interrogator resolution is another cause of distortion for the strain profile. In this MAPOD approach, its effect is considered by convolving a rectangular function (Equation (13)) with the DOFS strain profile:

(13) $$\Pi_{i}\left(x\right)=\left\{\begin{array}{l} \frac{1}{\Delta X},\;\left|x\right|\leq \frac{\Delta X}{2}\\ 0,\;\left|x\right|>\frac{\Delta X}{2} \end{array}\right.\;$$

where the symbol $\left(\Pi_{i}\right)$ denotes a rectangular function having a unitary area and width equal to the interrogator resolution $\left(\Delta X\right)$. Hence, the theoretical measured strain profile, $\varepsilon_{m}^{T}$, is given by Equation (14):

(14) $$\varepsilon_{m}^{T}\left(x\right)=\left(\Pi_{i}⊗\varepsilon_{f}\right)\left(x\right)$$

In Equation (14), the superscript $\left(T\right)$ highlights that the measurement is only theoretical, i.e., performed without noise.

### 2.4. Human Factors

#### 2.4.1. Hot-Touch Error

The first step in DOFS experiments is to locate a certain spatial coordinate in the optical fiber. Indeed, one must correlate the spatial frame of reference in the interrogator software with specific physical points in the DOFS. This is usually done by applying the so-called *hot touch*, a concentrated heat source, and reading the coordinate of the induced peak in the strain profile in the software. Ideally, the heat source should be infinitely narrow, but in reality, it is not, thus introducing an error in the coordinate locations along the fiber.

In this preliminary stage of the MAPOD framework, this uncertainty source is incorporated into the model using Equation (15) by shifting the x vector for each specimen by a quantity $\epsilon_{ht}$, leading to a new translated spatial domain vector ($x^{\prime }$):

(15) $$x^{\prime }=x+\epsilon_{ht}1_{N}\;$$

where $\epsilon_{ht}\;\sim \;N\left(0,\sigma_{ht}\right)$ is sampled from a normal distribution with a zero mean and a user-defined standard deviation ($\sigma_{ht}$), and $1_{N}$ is a vector of length N+1 of ones (Equation (16)):

(16) $$1_{N}=\left[1_{0},\ldots ,1_{N}\right]$$

#### 2.4.2. Bonding Error

The bonding error is the second type of human factor uncertainty source capable of affecting the system response. It is crucial to identify the start and the end of the bonded region in the DOFS. Here, an additional uncertainty source can be present, even assuming a perfect *hot*-*touch* procedure is accomplished. Indeed, bonding is never perfect, and the glue can infiltrate underneath the DOFS and thus extend the bonded region by a few millimeters. Moreover, this adhesive leakage, being an undesired effect, is often irregular, leading to unpredictable strain profiles in the transient region on the onset of bonding. Therefore, this uncertainty source must be incorporated into the model and strictly relates to the researcher’s expertise and the available equipment.

In the bonded region, the strain transfer is governed by the equations described in Section 2.2. On the other hand, the strain transfer is posed to zero in the regions outside the bonded area. The bonding error is simply taken into account by increasing or decreasing the bonded region size. In this MAPOD framework, the distance between the onset of the bonded region and the initial crack tip is defined by the d parameter as follows:

(17) $$d=d_{m}+\epsilon_{d}\;$$

where $d_{m}$ and $\epsilon_{d}$ are the mean and random components of d. Specifically, $\epsilon_{d}$ is sampled from a normal probability distribution with a zero mean and a $\sigma_{b\epsilon }$ bonding error standard deviation.

(18) $$\epsilon_{d}\;\sim \;N\left(0,\sigma_{b\epsilon }\right)\;$$

### 2.5. Environmental Noise

Noise is modeled by constructing a strain noise vector $\left(Z\right)$, defined as

(19) $$Z=\left[z_{1},\cdots ,z_{N}\right]\;$$

The ith element $\left(z_{i}\right)$ is defined as

(20) $$z_{i}\;\sim \;N\left(0,\sigma_{z}\right)\;$$

where $N\left(0,\sigma_{z}\right)$ denotes a normal distribution with a zero mean and a standard deviation equal to $\sigma_{z}$. One can assess $\sigma_{z}$ by performing a series of repeated measurements with no load applied to the specimen.

Finally, the real measured strain $\left(\varepsilon_{m}\right)$, which also takes into account the effect of noise, is obtained by simply superimposing the strain noise vector (**Z**) to the theoretically measured strain profile $\varepsilon_{m}^{T}$, as outlined in Equation (21):

(21) $$\varepsilon_{m}\left(x\right)=\varepsilon_{m}^{T}+Z\;$$

## 3. The DCB Case Study

Delamination is one of the most common and dangerous damage mechanisms in composite structures, and DCBs are representative structures of many different components. Thanks to well-defined standards describing the experimental procedure [43], DCBs are relatively easy to manufacture and test, which is crucial to validate the MAPOD model.

### 3.1. Experimental Setup

The DCB specimens are manufactured according to the ASTM D5528 standard [43] employing AS4 HexPly 8552^®^ unidirectional carbon prepreg [44]. The DOFS used in the study is a single-core optical fiber with ORMOCER^®^ coating [45]. The fiber is bonded on the surface of the specimen using a cyanoacrylate adhesive (ThreeBond 1742^®^ [46]). In the experiment, three DOFS segments are bonded on the top surface to augment the available data collected in the static test.

**Figure 2:**
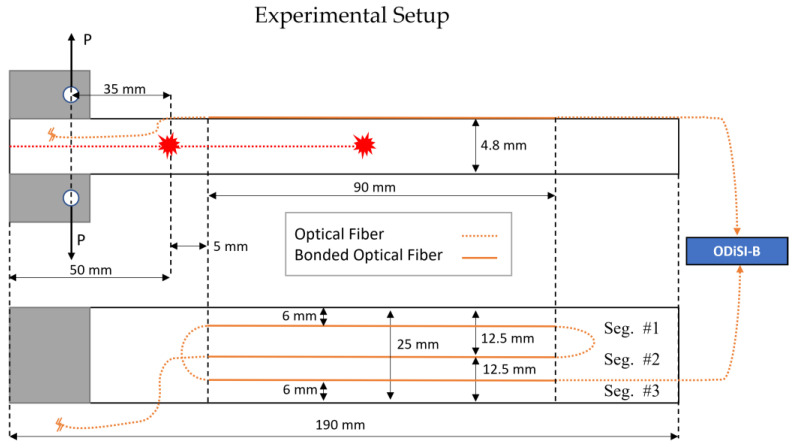
DCB scheme and DOFS layout for the static test (adapted from Falcetelli et al. [17]).

Figure 2 illustrates the specimen geometry and the relative DOFS installation layout used for the DCB static test.

The fibers are interrogated with the ODiSI-B [47] measuring system, which uses swept-wavelength coherent interferometry to estimate Rayleigh backscattering [48,49,50]. The spatial resolution and the sampling frequency of the sensing unit are 0.65 mm and 23.8 Hz, respectively. The DCB specimen is fixed on a Zwick—20 kN tensile test machine, and the load is applied with a displacement rate of 1 mm/min. The true crack length is estimated by exploiting its linear relationship with the cube root of the compliance value. The linear model is fitted by observing the delamination size from a 9-megapixel camera placed in front of the tensile test machine at different compliance values. Further details on the experimental setup can be found in Falcetelli et al. [17].

### 3.2. DCB Parametric Model

**Figure 3:**
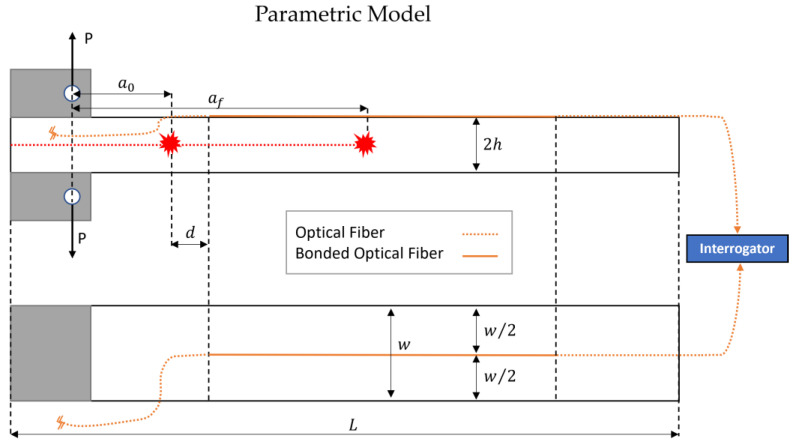
DCB scheme and DOFS layout of the proposed parametric model.

This section introduces a simplified DCB model, based on the experimental data derived in the previous section, to validate the proposed MAPOD methodology. Figure 3 describes the specimen geometry and the relative DOFS position.

In the parametric model, it is not necessary to consider three fiber segments on the DCB surface since the only limitation in the amount of synthetically generated data is the computational cost involved in the simulation. Therefore, it was decided to consider only a single DOFS segment bonded in the longitudinal direction at the center of the specimen to simplify the model.

The objective in this phase is to derive the strain distribution on the surface of the DCB specimen, $\varepsilon_{s}$, which is then fed into the strain transfer model.

Considering the two DCB arms as cantilever beams and referring to the Euler–Bernoulli theory, it is possible to compute the bending moment about the z-axis (perpendicular to the paper and pointing outward in Figure 3) as a function of the distance from the crack tip [51]:

(22) $$M_{z}=Px\;$$

Equation (22) holds for $x\;\in \;\left[0,\;a\right]$ since $M_{z}$ must be null after the crack tip at  x =a, where a denotes the crack length.

The applied load, P, decreases with the delamination length according to a function that can be determined experimentally or through FEM simulations [52]. In this case, $P\left(a\right)$ was determined by a third-degree polynomial regression (Equation (23)) using the experimental static test data of the DOFS with ORMOCER^®^ coating [17].

(23) $$P\left(a\right)=c_{0}+c_{1}a+c_{2}a^{2}+c_{3}a^{3}+\epsilon_{p}\;$$

The random component $\epsilon_{P}$ accounts for the impossibility of reproducing the exact loading condition across different specimens:

(24) $$\epsilon_{p}\;\sim \;N\left(0,\sigma_{p}\right)\;$$

where $\sigma_{p}$ represents the standard deviation of the normal distribution with a mean equal to zero. Its value can be assessed from the analysis of previous experimental activity or based on the expected operational loading conditions for a given application.

Applying Navier’s formula (Equation (25)) to the upper arm of the DCB shown in Figure 3, one can find the stress distribution along the x-axis, $\sigma_{x}$:

(25) $$\sigma_{x}=\frac{M_{z}}{I_{z}}y\;$$

where y is the spatial coordinate along the thickness direction, and $I_{z}$ denotes the moment of inertia about the *z*-axis (perpendicular to the paper pointing outward in Figure 3). The moment of inertia for a rectangular cross-section with a width and height equal to w and h, respectively, can be computed as follows:

(26) $$I_{z}=\frac{wh^{3}}{12}\;$$

Moreover, the specimen width and thickness could vary across the different specimens due to the variability in the manufacturing process. The variability can be estimated by measuring the specimens under test with a caliper. In this case study, the width and thickness variabilities were considered negligible compared to other uncertainty sources.

Finally, the strain on the DCB surface, $\varepsilon_{x}$, can be obtained by dividing the stress distribution along the *x*-axis, $\sigma_{x}$, by the flexural modulus $E_{x}$:

(27) $$\varepsilon_{x}=\frac{\sigma_{x}}{E_{x}}\;$$

The flexural modulus was computed using the following Equation (28), referring to the ASTM D5528 standard [43]:

(28) $$E_{x}=\frac{64{\left(a+\left|\Delta \right|\right)}^{3}}{Cw{\left(2h\right)}^{3}}\;$$

where C denotes the compliance and is defined as the ratio between the load point deflection, δ, and the applied load, P. On the other hand, Δ is a crack length correction used to account for the possible rotation at the crack tip. Its value corresponds to the abscissa where the least squares regression of the cube root of compliance, $\sqrt[{3}]{C}$, against the delamination length, a, is equal to zero.

Then, the expected strain distribution along the x-axis at the specimen surface is obtained by substituting the value of $\sigma_{x}$, obtained posing y=h/2 in Equation (25) into Equation (27):

(29) $$\varepsilon_{x}=\frac{M_{z}}{E_{x}I_{z}}\frac{h}{2}\;$$

### 3.3. Delamination Modeling from a MAPOD Perspective

The proposed MAPOD approach simulates delamination growth in a DCB specimen of length L, for a user-defined number of cracks, $n_{c}$, starting from a user-defined initial crack length, $a_{0}$, to a user-defined final crack length, $a_{f}$. It is assumed that every crack length measurement is taken after a certain delamination length increment, Δa. The true crack length, $a_{true}$, is usually visually estimated, thus introducing uncertainty in the model. Therefore, it is further assumed that Δa is composed of a mean constant value, $\Delta a_{m}$, and a random error, $\varepsilon_{a}$:

(30) $$\Delta a=\Delta a_{m}+\varepsilon_{a}\;$$

where $\varepsilon_{a}$ follows a normal probability distribution with a zero mean and a standard deviation $\sigma_{a}$:

(31) $$\varepsilon_{a}\sim \;N\left(0,\sigma_{a}\right)\;$$

The $\sigma_{a}$ value is challenging to assess and depends on many factors, such as the loading type, the geometry, and the material properties. Therefore, the best option is to evaluate $\sigma_{a}$ using data from a pilot study experiment.

On the other hand, $\Delta a_{m}$ can be computed as

(32) $$\Delta a_{m}=\frac{a_{f}-a_{0}}{n_{c}-1}\;$$

Then, the model generates a vector of crack lengths, a, which is defined in Equation (33):

(33) $$a=a_{m}+\varepsilon_{a}$$

where $a_{m}$ and $\varepsilon_{a}$ are the mean and random vectors of crack lengths defined in Equations (34) and (35), respectively:

(34) $$a_{m}=\left[a_{0},\;a_{0}+\Delta a_{m},\;\cdots ,\;a_{0}+n_{c}\Delta a_{m}=a_{f}\right]\;$$

(35) $$\varepsilon_{a}=\left[\varepsilon_{a1},\;\cdots ,\varepsilon_{an_{c}}\right]\;$$

In this case, the spatial domain, $x\;\in \;\left[0,\;L\right]$, is discretized with a user-defined spatial resolution, Δx, which must be sufficiently smaller than the average delamination increment, and thus it must be that $\Delta x\ll \Delta a_{m}$. This implies that the spatial domain vector, **x**, defined in Equation (1), has N+1 elements, with N=L/Δx.

## 4. Results

### 4.1. Model Validation

Table 1 shows the parameter configuration for the following demonstrative example, classifying the sources of uncertainty into within- and between-specimen variability (if the parameters are just model settings, they belong to neither of the two classes).

**Figure 4:**
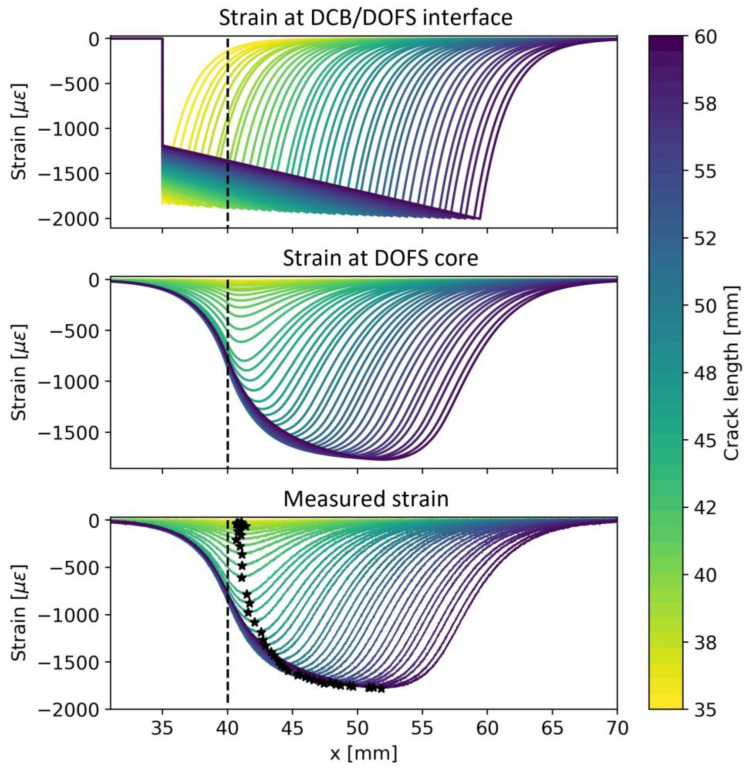
Simulation of strain profiles for different delamination lengths at the DCB surface (**top**), DOFS core (**middle**), and interrogator (**bottom**). The black star-shaped markers indicate the absolute minimum of the strain profile, where the crack tip is expected to be located.

Figure 4 shows the model outputs for the strain profile at the DOFS/DCB interface (top), the DOFS core (middle), and the interrogator-measured strain (bottom). Every line symbolizes the strain profile given at a specific crack length value, highlighted by the color bar on the right-hand side. The vertical dashed line shows the onset of the bonded region of the DOFS. It is possible to notice that since the DOFS is bonded 5 mm apart from the initial crack length, the fiber is not sensitive to damage at the initial phase of delamination growth. Once delamination reaches the bonded region and eventually grows underneath the DOFS, the measured strain profiles bend downward. The black star-shaped markers in the bottom subplot of Figure 4 indicate the absolute minimum of the strain profile, where the crack tip is expected to be located [32,53].

**Figure 5:**
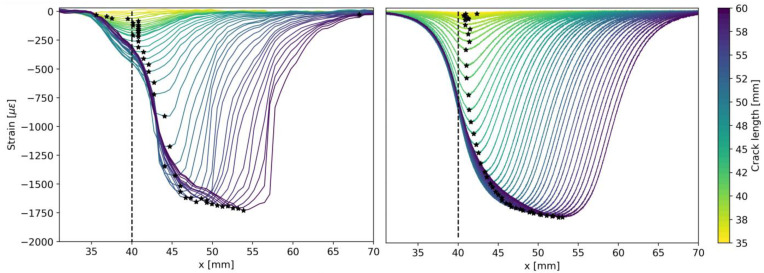
Measured strain profiles in DCB quasi-static test [17]: experimental data (**left**) and MAPOD predicted result (**right**). The black star-shaped markers indicate the absolute minimum of the strain profile, where the crack tip is expected to be located.

In Figure 5, experimental data (left) are compared with the results predicted by the model (right). From a qualitative perspective, it is possible to notice that experimental data show a higher degree of variability along the DOFS axis. Several factors can cause the difference. First, the proposed DCB model is still a simplified version of the real DCB, which would require a higher level of modeling to achieve more accurate results. For example, the model does not consider the possibility of having a curved crack front, which is common in DCB specimens. One might also note that the process zone can affect the strain at the specimen surface, but all these considerations are outside the scope of this paper. Second, the mechanical transfer function, Γ, used to predict the strain transfer does not vary with the x coordinate. This assumption neglects the presence of occasional defects in the bonding. These fluctuations in Γ might also be due to the intrinsic flaws present in the coating of the fiber.

The model validation continues through Section 4.1.1, where the experimental POD curve is compared with the MAPOD curve.

#### 4.1.1. Length at Detection Method and MAPOD Curve

As stated in the introduction section, the derivation of POD or MAPOD curves in SHM is substantially different from the NDE case due to the spatial and temporal correlation typical of SHM systems.

In NDE, every specimen returns a single point in the traditional $\hat{a}\;\mathrm{vs}.a$ or equivalent model: $\hat{a}\;\mathrm{vs}.\;a$, $\hat{a}\;\mathrm{vs}.\log\left(a\right)$, $\log\left(\hat{a}\right)\mathrm{vs}.\;a$, and $\log\left(\hat{a}\right)\mathrm{vs}.\;\log\left(a\right)$. In other words, every point corresponds to a measurement taken from a certain specimen, guaranteeing statistical independence. On the other hand, in SHM, every specimen returns a series of spatial- and time-correlated data since the permanently installed sensor continuously monitors the damage evolution.

Therefore, the application of the $\hat{a}\;\mathrm{vs}.\;a$ method (or the hit/miss method in the case of binary data), as initially conceived in the MIL-HKBK-1823A [12] for NDE applications, would lead to inconsistent results. Among the available statistical methods to handle SHM data, the LaD offers an intuitive and easy-to-implement approach. The alternative REM-based methodology has the advantage of using data more efficiently but at the expense of increasing the complexity of the algorithm and the computational burden [15]. Therefore, in this preliminary development of the MAPOD framework for DOFSs, the LaD is selected as the most appropriate approach.

In the LaD method, a certain damage index (DI) is plotted against a certain damage-related feature, in this case, the delamination length. However, differently from the traditional $\hat{a}\;\mathrm{vs}.\;a$ approach, the procedure is repeated for every specimen, leading to a number of regression lines equal to the number of specimens considered in the analysis.

The LaD method can handle spatial and temporal correlated data because it considers only the measurements in correspondence with the first detection, which is the intersection of every line with the user-defined threshold. Therefore, the threshold line contains a population of crack/delamination lengths at detection which is usually assumed to follow a normal or log-normal probability distribution. Therefore, assuming that the population is normally distributed, it is possible to compute the POD curve as follows:

(36) $$\mathrm{POD}\left(a\right)=\Phi_{norm}\left(\frac{a-\bar{x}}{s}\right)\;$$

where $\Phi_{norm}$ denotes the standard normal distribution cumulative distribution function, and $\bar{x}$ and s are the sample mean and standard deviation, respectively.

The lower confidence bound can be computed with the one-sided tolerance interval (OSTI) approach [54]:

(37) $$T=\bar{x}+K_{N,\gamma ,\alpha }\cdot s\;$$

where T and $K_{N,\gamma ,\alpha }$ are the tolerance interval and factor, respectively. The tolerance factor depends on the sample size (in this case, the number of specimens, N), the confidence level $\left(\gamma \right)$, and the detection level $\left(1-\alpha \right)$. A standard procedure to compute its value exploits the properties of the non-central *t*-distribution [15].

The LaD method requires defining a proper DI. The DI definition depends on the sensor technology and the type of damage to be monitored. For example, in certain cases, to obtain a DI, one can leverage multi-sensor data fusion [55,56] or more complex strategies based on deep learning [57]. In this study, involving delamination monitoring using DOFSs, it is logical to consider some damage-induced strain feature as DI. The most straightforward choice is to define the DI related to a certain crack length as the strain value recorded at the absolute minimum of each strain profile. Referring to Figure 5, this translates into considering the strain values in correspondence with the black star-shaped markers as a DI.

Then, one can take the absolute value and obtain a monotonically increasing DI as described by Equation (38):

(38) $$\;DI\left(a\right)=\ln\left|min\left[\varepsilon_{m}\left(a\right)\right]\right|\;$$

**Figure 6:**
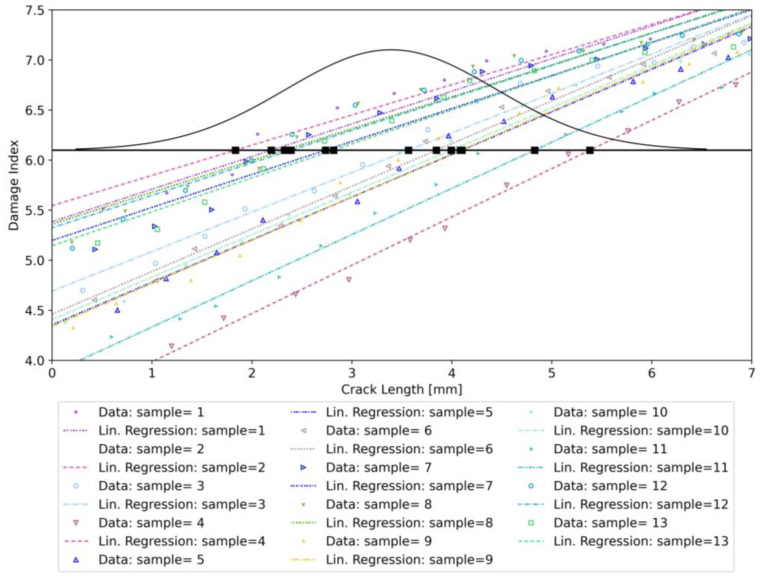
Model-Assisted LaD method applied to synthetic DOFS data for crack detection.

For validation purposes, the same threshold value was used as in the previous experimental study [17] of $\exp\left(6.1\right)\;\mu \varepsilon$. Applying the LaD method using the DI defined in Equation (38) returns the plot shown in Figure 6.

Every regression line is associated with a different specimen and has its own slope and intercept, reflecting between-specimen variability. At the same time, data belonging to the same specimen are scattered around the mean value represented by the regression line, showing within-specimen variability (see Table 1).

**Figure 7:**
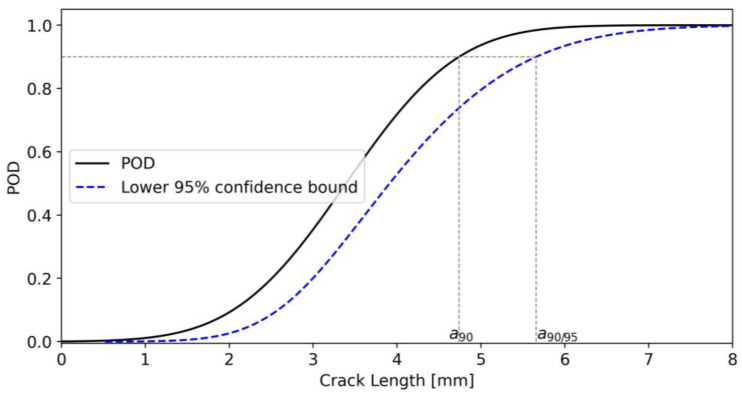
POD and its lower 95% confidence bound for simulated data.

Assuming that the crack lengths at detection are normally distributed, it is possible to obtain the corresponding POD curve (see Figure 7) by computing the cumulative function of the LaD distribution. The distance of the lower 95% confidence bound (blue dashed line) from the POD curve (solid black line) reflects system uncertainty. High uncertainty values in the model parameters will result in a lower 95% confidence bound far away from the original POD curve.

**Figure 8:**
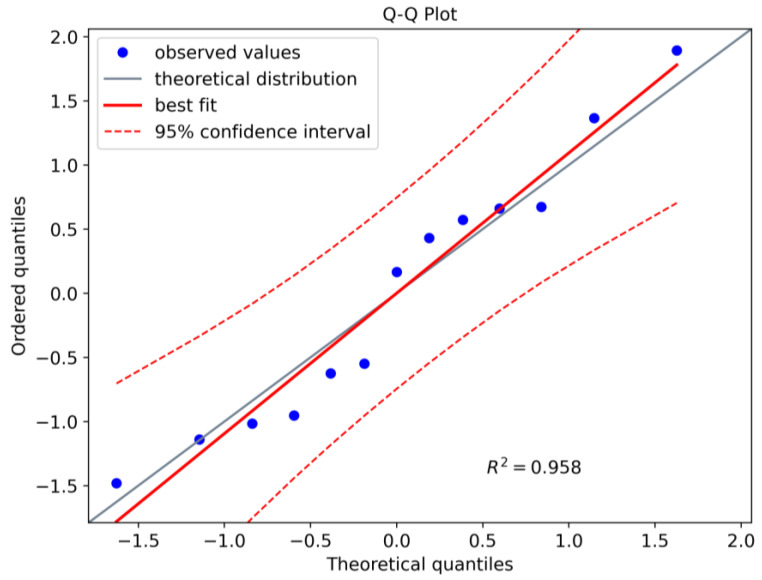
Normality Test for the LaD method.

One can verify the normality assumption of the LaD method with the so-called Anderson–Darling test (Figure 8). The null hypothesis $\left(H_{0}\right)$ assumes that the data are normally distributed and should be rejected if, for a given significance level $\left(\alpha \right)$, the critical value is lower than the Anderson–Darling statistics (A^2^). With α=0.05 and N=13, the critical value equals 0.679. The A^2^ statistics for the simulated LaD data plotted is 0.329. Thus, according to the normality test, $H_{0}$ should not be rejected and the crack lengths at detection can be considered normally distributed.

Finally, Table 2 compares the experimental and MAPOD values for $a_{90}$ (the crack length value having a 90% probability of being detected) and $a_{90/95}$ (the crack length value having a 90% probability of being detected with 95% confidence). The relative error for $a_{90}$ is −3.87 %, whereas the relative error for $a_{90/95}$ is +1.76 %, demonstrating the accuracy of the proposed model.

### 4.2. Sensitivity Analysis

In this section, the objective is to perform a preliminary qualitative analysis of the effect of the most important variables on the $a_{90}$ and $a_{90/95}$ generated through the MAPOD curves. The threshold definition is crucial in this analysis and can significantly affect the result. The threshold value must guarantee a constant probability of false alarm (PFA). In the literature, no standard methodology defines the threshold for the LaD method. Here, the threshold was defined according to Equation (39):

(39) $$th=\max\left(\varepsilon_{m}^{j}\left(x=d\right)\right)+3\sigma_{z};\;j=1,\cdots ,N$$

where, $\varepsilon_{m}^{j}\left(x=d\right)$ represents the vector containing the measured strain values at the onset of the bonded region for each specimen. Therefore, th is chosen by adding three noise standard deviations to the highest measured strain values between the tested samples at the onset of the bonded region. This definition is noise-dependent, which is coherent with maintaining a constant PFA, and prevents the possibility of obtaining negative crack lengths at detection.

Table 3 shows the parameter settings used in the sensitivity analysis. The data structure 〈−|−|−〉 symbolizes that the parameter is linearly swept from an initial value (first field) to a final value (second field) on a logarithmic scale with a certain number of elements (third field). For example, $\mu_{\lambda }=\langle 0.001|20|100\rangle$ means that $\mu_{\lambda }$ is linearly swept on a logarithmic scale from 0.001 mm to 20 mm considering 100 elements.

#### 4.2.1. Noise Effect (Case 1)

**Figure 9:**
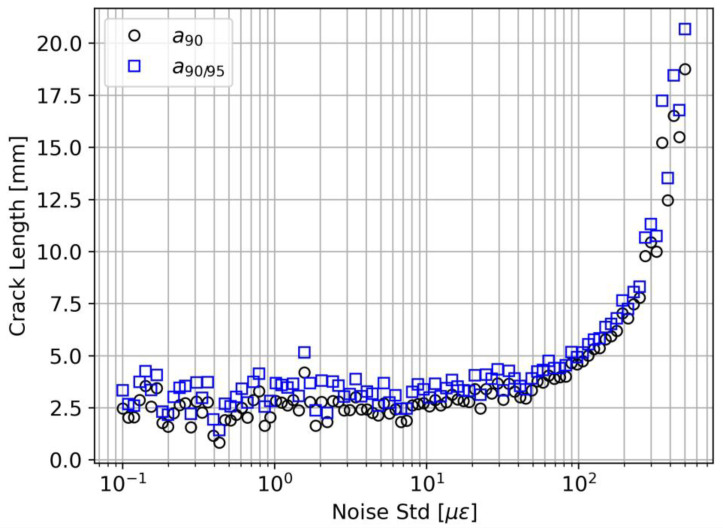
Noise effect on $a_{90}$ and $a_{90/95}$.

Figure 9 illustrates the effect of measurement noise on the detection performance. The results show that the effect of noise is observable only for standard deviation values higher than 50 με. This result might seem counterintuitive since 50 με is a relatively high value for common applications, and one would expect a degradation of the performance starting from lower values of $\sigma_{z}$. However, to comprehend the result, one must distinguish between detection and localization performance and how the DI and the threshold value are defined. Indeed, in terms of localization, the performance is compromised even at lower $\sigma_{z}$ values, because the minimum peaks in the strain profile would be scattered along the fiber length and mainly attributed to noise. However, the system is much more robust in terms of crack detection. Indeed, the DI (see Equation (38)) considers only the strain value in the minimum of a given strain profile, not its location. This definition allows the model to find a relation between the DI and a even in particularly noisy conditions. The limit of 50 με depends on the specific application of and especially on the damage-induced strain. In this DCB case study, below 50 με, the SNR is still high enough to make a detection. However, if the damage-induced strain is low, the effect of noise would be much more disruptive even at lower values. It is important to highlight that the threshold definition is of paramount importance. According to Equation (39), the threshold increases with $\sigma_{z}$, and this guarantees a constant PFA. With a fixed threshold value, we would witness the paradoxical and fictitious improvement of the detection performance caused by a corresponding increase in the PFA.

#### 4.2.2. Strain Transfer Effect (Case 2)

**Figure 10:**
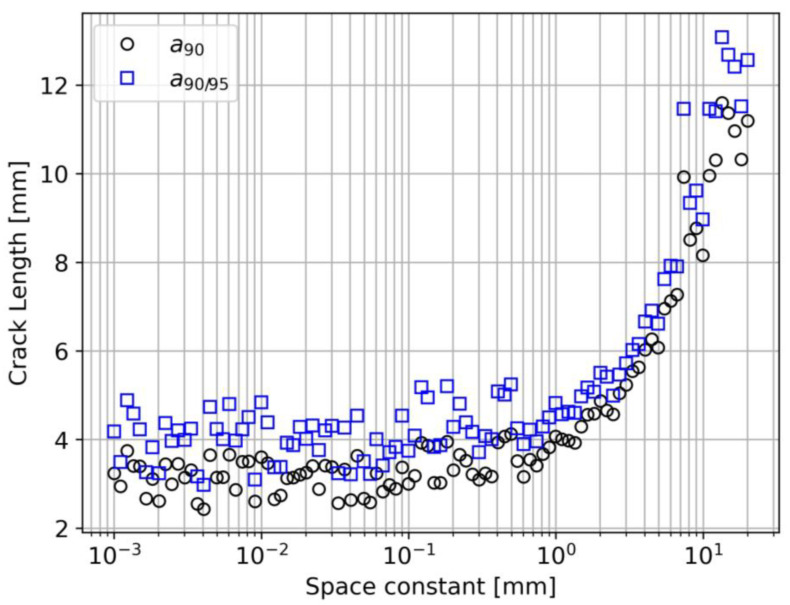
Space constant effect on $a_{90}$ and $a_{90/95}$.

The shear lag constant effect on the detection capabilities is highlighted in Figure 10. When 0.001 mm<λ<1 mm, $a_{90}$ and $a_{90/95}$ show a steady value of around 4 mm. Then, for λ>1 mm, it is possible to observe a sudden increase in $a_{90}$ and $a_{90/95}$. This means that stiffer DOFSs, characterized by low λ values, have better detection performance, but the benefit is negligible for λ values lower than 1 mm.

This analysis can also be used to implement a degradation model of the DOFS coupling with the structure. Indeed, different values of λ can correspond to different adhesives but also subsequent moments in time of the same adhesive undergoing aging.

#### 4.2.3. Interrogator Resolution Effect (Case 3)

**Figure 11:**
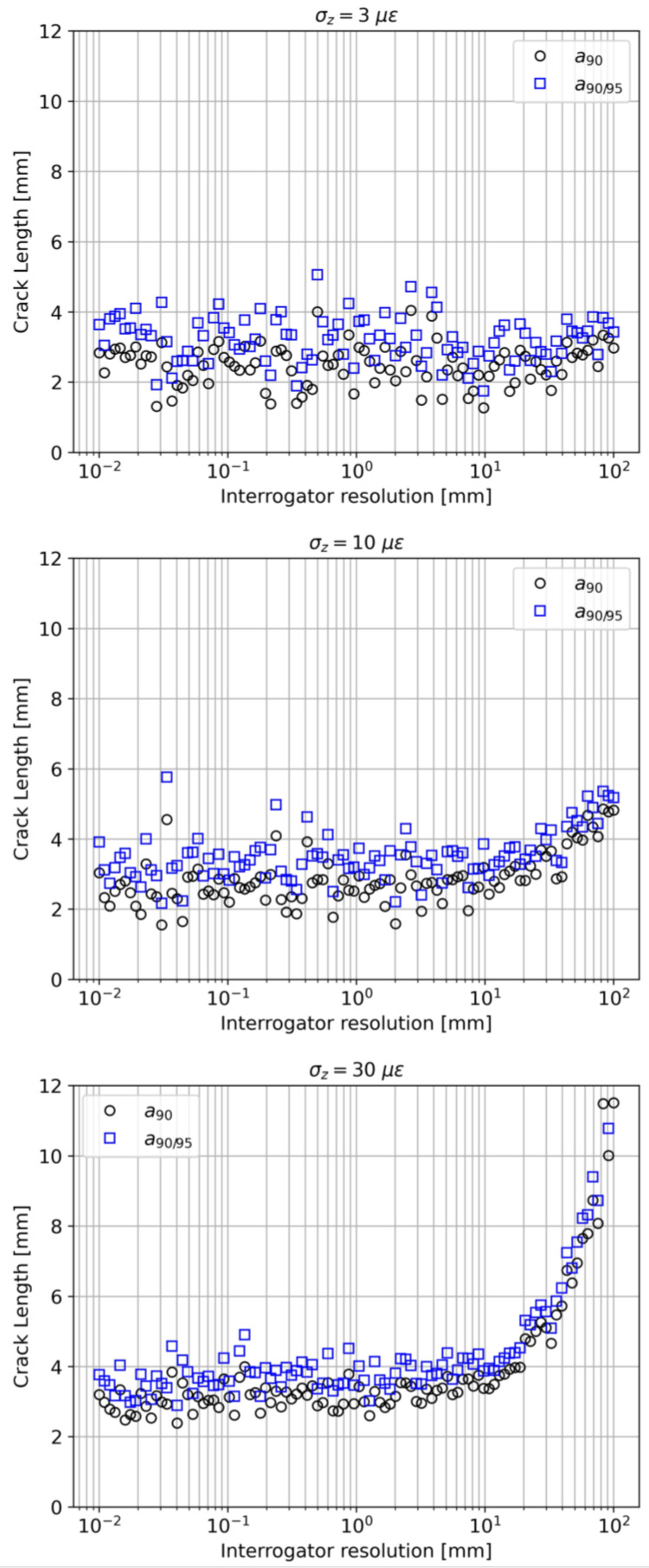
Interrogator resolution effect on $a_{90}$ and $a_{90/95}$, at $\sigma_{z}=3\;\mu \varepsilon$ (**top**), $\sigma_{z}=10\;\mu \varepsilon$ (**middle**), and $\sigma_{z}=30\;\mu \varepsilon$ (**bottom**).

Figure 11 is composed of three subplots, representing the effect of the interrogator resolution Δx at three different noise levels: $\sigma_{z}=3\;\mu \varepsilon$, $\sigma_{z}=10\;\mu \varepsilon$, and $\sigma_{z}=30\;\mu \varepsilon$. In principle, an ideal interrogator with an infinite spatial resolution (Δx → 0) should provide the best performance. Figure 11 confirms this belief but only to a certain extent, depending on the noise level. At low noise levels ($\sigma_{z}=3\;\mu \varepsilon$), the resolution effect on $a_{90}$ and $a_{90/95}$ is negligible. Indeed, even if a poor resolution flattens the measured strain profile, the SNR is still high enough to ensure comparable detection performance. Once again, similarly to what emerged in Section 4.2.1, one must distinguish detection performance from localization performance. At a higher noise level ($\sigma_{z}=10\;\mu \varepsilon$), the effect of the interrogator resolution is observable. Finally, as the noise level increases even further ($\sigma_{z}=30\;\mu \varepsilon$), the critical SNR is reached at even lower values of Δx. This kind of simulation can be beneficial to the engineer in selecting the right interrogator in the preliminary design phase of the SHM system. Indeed, considering all the available information about the other model parameters, the SHM system equipped with such an interrogator should satisfy specific requirements for $a_{90}$ and $a_{90/95}$.

#### 4.2.4. Hot-Touch Error Effect (Case 4)

**Figure 12:**
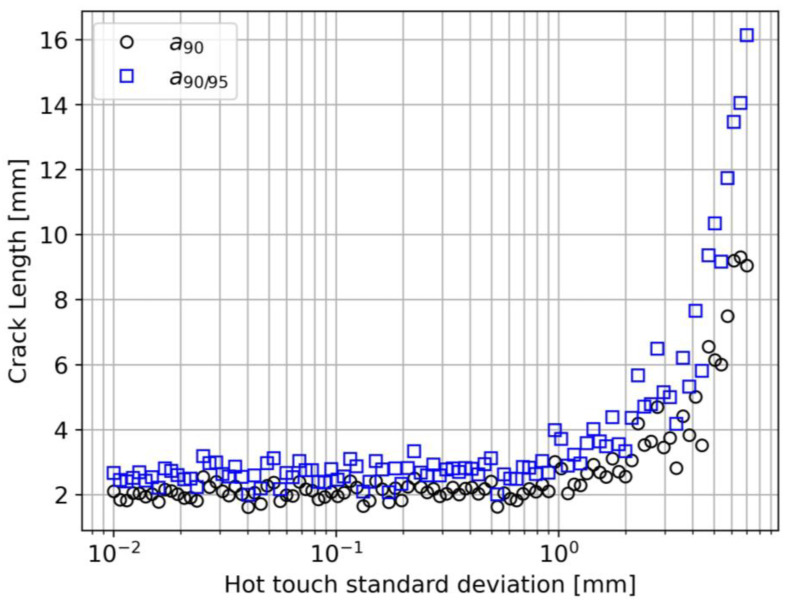
Interrogator resolution effect on $a_{90}$ and $a_{90/95}$.

The hot-touch standard deviation reflects one of the human factor effects on the detection performance. It is important to point out that in a noisy environment, performing a satisfactory hot-touch procedure is extremely difficult. Hot-touch standard deviation values greater than 1 mm in real operational conditions are relatively common. However, Figure 12 suggests that $\sigma_{ht}$ can deeply change the detection performance of the system starting from $\sigma_{ht}=1\;\mathrm{mm}$. As pointed out for the previous cases, this value can change depending on the other model parameters, but it is fundamental to notice how a procedure that is often considered of minor importance can have a considerable impact on $a_{90}$ and $a_{90/95}$.

#### 4.2.5. Bonding Error Effect (Case 5)

**Figure 13:**
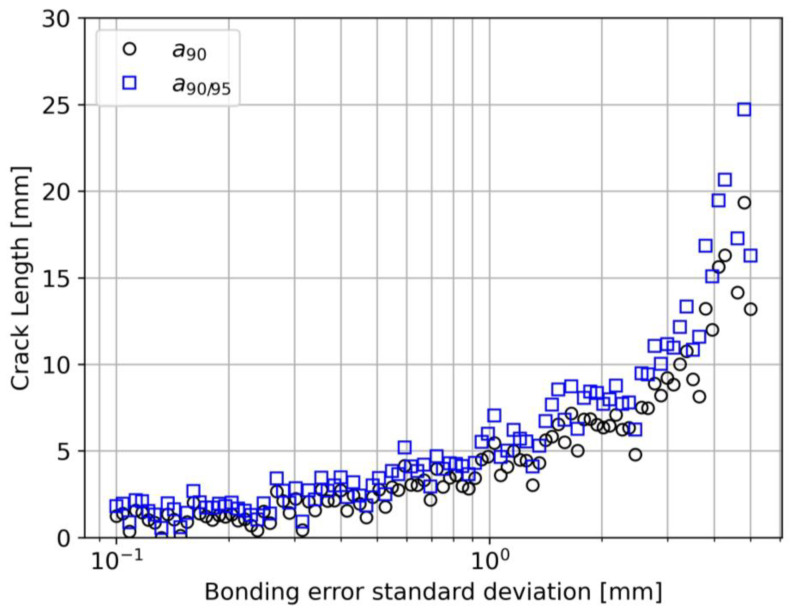
Bonding error standard deviation effect on $a_{90}$ and $a_{90/95}$.

The bonding error is a second possible human factor issue that can potentially affect the performance of the system. The semi-logarithmic plot illustrated in Figure 13 shows that a lack of repeatability in the bonding procedure could lead to a detrimental effect. It is important to notice that this deterioration of the detection performance can start already at relatively low values of $\sigma_{b}$, even lower than 1 mm. The result is somewhat expected since an error in the starting point of the bonded region would inevitably anticipate or delay the strain transfer process. As a consequence, data will be more scattered, the threshold will increase, and the result POD curve will produce higher $a_{90}$ and $a_{90/95}$ values.

## 5. Discussion

### Upscaling of the MAPOD Methodology to a Real Aerospace Structure

From a practical perspective, assessing the upscaling capabilities of the presented MAPOD framework is crucial. In other words, it is essential to understand whether it is possible to transpose the MAPOD curves to a higher structural component in a building block approach.

Referring to the flowchart of the proposed MAPOD approach (see Figure 1), one can notice that the interrogator block receives as input the results of the strain transfer block, and the strain transfer block receives as inputs the outputs of the structure/damage block. Therefore, upscaling the presented MAPOD framework to a more complex and representative structural item would impact only the first block, which is highly desirable.

Therefore, applying the presented MAPOD framework to a more realistic case implies substituting the first block (DCB model described in Section 3) with another block representing the new structure.

For example, debonding and delamination can occur in stiffened panels at the skin–stringer interface or between adjacent layers having different fiber orientations [58]. In this case, one could directly model the stiffened composite panel using finite element analysis (FEA) or by whatever model that can return as outputs the strain values at the DOFS/ structure interface in the presence of damage.

Several studies have investigated debonding and delamination in stiffened composite panels for aerospace applications in a damage tolerance scenario with DOFSs [59,60] and FBGs [61], considering damage sizes in the 40–80 mm range. In this study, it is shown that in a DCB specimen, it is possible to achieve $a_{90/95}$ of 5.66 mm (see Table 2). However, a direct transposition of this result to a full-scale composite panel would be misleading. Indeed, in a skin–stringer debonding scenario, delamination can occur at different mode-mixity [62,63], and the DOFS sensitivity can differ for different opening modes.

A solution would be developing MAPOD curves at a specimen level for different opening modes and opening mode-mixity. Then, using a building block approach [64], one could transpose the results to a higher-level component, selecting the MAPOD curve obtained in the scenario that better approximates the expected strain field near the damaged location.

The authors believe that upscaling POD and MAPOD curves is crucial for concrete and sustainable deployment of SHM in primary composite structures and certainly deserves further investigation.

## 6. Conclusions

This study focuses on the development of a MAPOD framework for DOFSs. This framework has never been established for DOFSs, only for a few SHM technologies such as GLW, bulk-wave ultrasonic sensors, and FBGs. From a methodological perspective, the modeling was divided into three blocks: the structure and damage model, the strain transfer model, and the sensing unit model. Each block receives as input several uncertainty sources, which were analyzed and modeled. The model was validated with previously collected data on an equivalent case study, showing comparable $a_{90}$ and $a_{90/95}$ values. The results of the sensitivity analysis highlight the main variables affecting POD curves and, specifically, $a_{90}$ and $a_{90/95}$. These variables are the noise, the strain transfer space constant, the interrogator resolution, and the human factors (hot-touch and bonding errors).

Table 4 summarizes the sensitivity analysis results. For each case, it is shown the value obtained in correspondence with $a_{90/95}$ equal to 10 mm and in correspondence with a 10% raise (↑↑) of $a_{90/95}$ with respect to its initial steady state value. Assuming that 10 mm is the maximum allowed delamination size for a given application, the first value can be considered the critical parameter threshold that should not be exceeded. On the other hand, the second value symbolizes the threshold below which the parameter does not affect the MAPOD curve. In other words, any effort to reduce its value does not produce any improvement in terms of $a_{90/95}$.

The impact of noise on damage detection is negligible until the SNR falls below a critical value. The MAPOD approach for the selected case study showed that the system is robust against noise standard deviation values below approximately 50 με. The strain transfer analysis highlighted that $a_{90}$ and $a_{90/95}$ significantly increase for space constant $\left(\lambda \right)$ values higher than approximately 1 mm. This result is the first of its kind since the strain transfer problem has never been analyzed in an SHM damage detection probabilistic framework. The effect of the interrogator resolution $\left(\Delta x\right)$ was negligible for low values of noise $\left(\sigma_{z}=3\;\mu \varepsilon \right)$, but its effect has been observed for higher noise values $\left(\sigma_{z}=10\;\mu \varepsilon \;\mathrm{and}\;\sigma_{z}=30\;\mu \varepsilon \right)$. The human factors, namely the hot-touch and bonding errors, were also analyzed. These kinds of uncertainty sources have never been analyzed before this research and were revealed to be key variables with a significant impact on $a_{90}$ and $a_{90/95}$.

This research has unfolded only some aspects of SHM systems based on DOFSs applied to composite structures undergoing mode I static loading conditions. The model should be further refined to model even more uncertainty sources, such as a variable strain transfer function along the optical fiber length, or to simulate other loading conditions (such as fatigue) or crack opening modes (mode II and mode III).

The proposed approach could be used for modeling changing EOCs and simulating their effect on POD curves. For instance, increasing the strain transfer constant λ in time would reproduce a degradation of the strain transfer properties expected with the aging of the adhesive or the optical fiber coating.

This MAPOD framework appears promising to study not only detection but also localization and sizing problems. The extension of this approach to the subsequent steps of the SHM paradigm would lead to the development of the model-assisted probability of localization and sizing (MAPOL and MAPOS). The application of the presented approach to MAPOL and MAPOS will be the subject of future studies.

## Figures and Tables

**Table 1:** MAPOD parameter setting.

Variable	Value	Within-Specimen Variability	Between-Specimen Variability
N	13	No	No
$L\;\left[\mathrm{mm}\right]$	135	No	No
$h\;\left[\mathrm{mm}\right]$	2.4	No	No
$w\;\left[\mathrm{mm}\right]$	25	No	No
$n_{c}$	20	No	No
$a_{0}\;\left[\mathrm{mm}\right]$	35	No	No
$a_{f}\;\left[\mathrm{mm}\right]$	48	No	No
$\sigma_{a}\;\left[\mathrm{mm}\right]$	0.05	Yes	No
$\sigma_{z}\;\left[\mu \varepsilon \right]$	3	Yes	No
$\mu_{\lambda }\;\left[\mathrm{mm}\right]$	1.7	No	Yes
$\sigma_{\lambda }\;\left[\mathrm{mm}\right]$	0.17	No	Yes
$\Delta x\;\left[\mathrm{mm}\right]$	0.65	No	No
$\sigma_{ht}\;\left[\mathrm{mm}\right]$	1	No	Yes
$\sigma_{b\epsilon }\;\left[\mathrm{mm}\right]$	0.5	No	Yes

**Table 2:** Comparison of experimental and simulated $a_{90}$ and $a_{90/95}$.

Variable	Experiment	MAPOD	Relative Error (%)
N	13	13	
$a_{90}\;\left[\mathrm{mm}\right]$	4.93	4.74	−3.87
$a_{90/95}\;\left[\mathrm{mm}\right]$	5.56	5.66	+1.76

**Table 3:** Parameter setting for the sensitivity analysis.

Variable	Case 1	Case 2	Case 3	Case 4	Case 5
N	20	20	20	20	20
$L\;\left[\mathrm{mm}\right]$	135	135	135	135	135
$h\;\left[\mathrm{mm}\right]$	2.4	2.4	2.4	2.4	2.4
$w\;\left[\mathrm{mm}\right]$	25	25	25	25	25
$n_{c}$	20	20	20	20	20
$a_{0}\;\left[\mathrm{mm}\right]$	35	35	35	35	35
$a_{f}\;\left[\mathrm{mm}\right]$	48	48	48	48	48
$\sigma_{a}\;\left[\mathrm{mm}\right]$	0.05	0.05	0.05	0.05	0.05
$\sigma_{z}\;\left[\mu \varepsilon \right]$	〈0.1|500|100〉	3	$\left[3,\;10,\;30\right]$	3	3
$\mu_{\lambda }\;\left[\mathrm{mm}\right]$	1.7	〈0.001|20|100〉	1.7	1.7	1.7
$\sigma_{\lambda }\;\left[\mathrm{mm}\right]$	0.17	0.17	0.17	0.17	0.17
$\Delta x\;\left[\mathrm{mm}\right]$	0.65	0.65	〈0.01|100|100〉	0.65	0.65
$\sigma_{ht}\;\left[\mathrm{mm}\right]$	1	1	1	〈0.01|7|100〉	1
$\sigma_{b\epsilon }\;\left[\mathrm{mm}\right]$	0.5	0.5	0.5	0.5	〈0.1|5|100〉

**Table 4:** Summary of the sensitivity analysis results.

Condition	$\sigma_{z}\;\left[\mu \varepsilon \right]$	$\mu_{\lambda }\;\left[mm\right]$	$\Delta x\;\left[mm\right]$	$\sigma_{ht}\;\left[mm\right]$	$\sigma_{b\epsilon }\;\left[mm\right]$
$a_{90/95}=10$	117.4	7.2	[>100, >100, 92.4]	5.1	2.7
$a_{90/95}↑↑10\%$	49.9	0.9	[>100, 41.1, 13.5]	0.9	0.3

## Data Availability

Not applicable.
